# Hospital nurse managers' perspectives of the Magnet Recognition Program using an importance‐performance analysis: A quantitative cross‐sectional study

**DOI:** 10.1002/nop2.70015

**Published:** 2024-08-21

**Authors:** Eunha Ryoo, Seok Hee Jeong, Na Yeon Shin, Soyoung Yu

**Affiliations:** ^1^ Department of Nursing Dongnam Health University Suwon Republic of Korea; ^2^ College of Nursing Research Institute of Nursing Science Jeonbuk National University Jeonju‐si Republic of Korea; ^3^ CHA University‐Bundang CHA Medical Center Seongnam‐si Republic of Korea; ^4^ College of Nursing CHA University Pocheon Republic of Korea

**Keywords:** importance–performance analysis, magnet accreditation, nurse administrator

## Abstract

**Aim:**

To explore the perspectives of nursing managers in Korean hospitals on the Magnet Recognition Program using importance–performance analysis.

**Design:**

A descriptive quantitative cross‐sectional design with a survey methodology was used to evaluate nursing managers' perceptions of the Magnet Recognition Program criteria.

**Methods:**

After the Magnet Recognition Program's content validity was confirmed, an online survey was administered to 150 nursing managers from 10 hospitals. The results were analysed using importance–performance analysis.

**Results:**

The average importance of the questionnaire for the developed Magnet Recognition Program criteria was 3.19 ± 135 and the performance was 2.90 ± 222. Items corresponding to the areas ‘Concentrate here’, ‘Keep up the good work’, ‘Possible overkill’ and ‘Low priority’ were identified using two importance–performance analysis frames. The items corresponding to ‘Concentrate here’ included evidence‐based nursing practice, the nursing professional practice model, nurses' participation in improving turnover rate and cases of innovation in nursing.

**Conclusion:**

This study highlights areas for improvement within the Magnet Recognition Program as perceived by Korean nursing managers, emphasizing evidence‐based practice, professional models and nurses' involvement in turnover reduction and fostering innovation.

**Public Contribution:**

To achieve Magnet recognition, hospitals must understand nursing managers' perspectives on the Magnet Recognition Program criteria. This study provides insights into enhancing the work environment for nurses in South Korean hospitals and lays the groundwork for developing effective Magnet certification programs. Introducing the Magnet program into South Korean hospitals may improve the overall nursing work environment and mitigate the serious problem of nursing staff turnover.

**Reporting Method:**

The findings were reported using the Strengthening the Reporting of Observational Studies in Epidemiology (STROBE) checklist.

## 1 BACKGROUND

The Magnet Recognition Program, accredited by the American Nurses Credentialing Center (ANCC), is a distinguished acknowledgment that mirrors exceptional nursing practices and high‐quality outcomes within a cultural framework of nursing care, professionalism and respect (Melnyk et al., 2020; Urden et al., 2021). Magnet designation status indicates excellence in nursing as it requires hospitals to adopt evidence‐based practice (EBP) and ensure clinical nurse involvement in research and EBPs (Nelson‐Brantley et al., 2020).

Since the Magnet Recognition Program was established in 1990, it has sought to provide a roadmap to achieving nursing excellence (Abuzied et al., 2022). The Magnet designation recognizes hospitals with excellent work environments for nurses and high‐quality patient care (Lasater et al., 2019). According to the ANCC (American Nurses Credentialing Center), Magnet hospitals have a greater percentage of satisfied nurses, lower turnover of registered nurses, fewer vacancies, higher patient satisfaction and better clinical results (Stone et al., 2019).

Hospitals that achieve Magnet recognition appear to have better outcomes for nurses (i.e., better job satisfaction, lower burnout and intention to leave) and patients (i.e., lower rates of mortality, failure to rescue, falls, hospital‐acquired infections and pressure ulcers) and achieve better performance measures (i.e., value‐based purchasing) (Hamadi, Borkar, et al., 2021; Hamadi, Martinez, et al., 2021; Kutney‐Lee et al., 2015; Lasater et al., 2017; Rodríguez‐García et al., 2020). A comprehensive review of these research results shows that medical institutions should strive to achieve Magnet designation.

The Magnet model (ANCC, n.d.‐a) comprises five core components (transformational leadership; structural empowerment; exemplary professional practice; new knowledge, innovations and improvements and empirical outcomes). Hospitals must pay a fee to obtain Magnet designation, and they must meet a range of standards in various areas to receive certification, including quality of leadership, organizational structure, management style, personnel policies and programs, professional models of care, quality of care, quality improvement, consultation and resources, autonomy, community and the hospital, nurses as teachers, nursing image, interdisciplinary relationships and professional development (Abuzied et al., 2022; Shin, 2019).

As of December 2023, 9.4% of the hospitals in the U.S. had been designated as Magnet‐certified hospitals, and 591 facilities had been designated worldwide, including in Saudi Arabia, the United Kingdom, Australia and Canada (ANCC, n.d.‐b). As of February 2024, South Korea (hereafter, Korea) had no designated hospitals, although many hospitals in the United States and internationally were pursuing Magnet designation or re‐designation (ANCC, n.d.‐a).

According to the results of a 2022 survey on Korean health and medical personnel, South Korean hospitals averaged 37 working hours and 117.21 patients per week (including outpatients and inpatients) and the two most common causes of job difficulties were ‘lack of pride as a professional’ and ‘lack of expertise and technology’. Additionally, in terms of work satisfaction, the items ‘wage level’, ‘personnel promotion and labour management’, ‘welfare’ and ‘labour intensity’ received low scores, and the percentage of nurses with turnover experience was 52.8%. The main reasons for nurses' turnover were ‘low compensation level’ (41.4%) and ‘heavy workload’ (40.8%) (Ministry of Health and Welfare, 2022). Moreover, despite various nursing policies, the resignation rates of Korean hospital nurses were 14.4% and 15.8% in 2021 and 2022, respectively. The resignation rate among new nurses significantly increased from 38.1% in 2017 to 52.8% in 2022 (Hospital Nursing Association, 2022). Nurses comprise the majority of healthcare institution staff, especially in medical institutions that rely heavily on human resources (Park & Park, 2023), and responding to high turnover is critical to achieving organizational goals. The international recognition and reputation that comes with a Magnet designation creates a set of standards that require an improved nurse‐working environment; therefore, it would be beneficial for Korean hospitals to introduce Magnet certification to reduce nurse turnover rates.

To obtain Magnet designation, the nurses' working environment must be improved. To do so, nursing managers' perspectives regarding the Magnet Recognition Program must be understood as managers play a crucial role in improving the working environment of their nursing staff. Currently, studies on Magnet‐designated hospitals have been conducted in various countries (Fischer & Nichols, 2019; Hamadi, Borkar, et al., 2021; Hamadi, Martinez, et al., 2021; Rodríguez‐García et al., 2020; Schlak et al., 2021; Stone et al., 2019); however, most research in Korea has only been cited indirectly (Choi & Seo, 2020; Lee & Park, 2021). Therefore, practical research on Magnet hospitals in Korea should be conducted. Most importantly, it is necessary to fully understand the perspectives of potential Magnet hospital nurse managers in Korea as the country is currently experiencing a serious shortage of skilled nurses and high turnover rates.

This study emphasizes the importance and performance of the Magnet Recognition Program, as recognized by nursing managers in Korean hospitals, and uses importance–performance analysis (IPA) to identify specific areas in need of focus. It aims to lay the foundation for improving the working environment of nurses in hospitals in Korea by evaluating nursing managers' perceptions of each Magnet designation standard using the IPA. The IPA was first proposed by Martilla and James (1977) and is used to evaluate the relative importance of various factors in a system or process and identify areas for improvement. It is widely considered an efficient approach for identifying and prioritizing points that require improvements in service processes (Xu et al., 2023; Zarei et al., 2020).

This study has two purposes. First, it provides a theoretical basis for effectively preparing for Magnet designation by evaluating the importance that nursing managers attach to Magnet recognition criteria to identify areas requiring improvement. Second, it actively introduces a Magnet‐designated hospital program in Korea and provides data needed to create an environment in which professional nurses can provide their patients with continuous high‐quality care. In summary, this study aims to establish a foundation for potential Magnet‐designated hospitals by confirming the IPA of each Magnet‐designated standard for Korean nurse managers. A comparative analysis of the importance and performance to confirm nurse administrators' degree of perception of Magnet recognition programs is especially useful for easily identifying areas for improvement.

## 2 METHODS

### 2.1 Study design

We used a descriptive quantitative cross‐sectional design with a survey methodology to enable nursing managers to evaluate the importance and performance of the Magnet Recognition Program criteria, which is a valuable and cost‐effective method for determining the relationships between variables (Polit & Beck, 2021).

### 2.2 Samples and settings

This study targeted nursing managers working in 10 hospitals in Korea who understood the purpose of the study and voluntarily agreed to participate. We used G*power 3.1.9.7 to select the appropriate sample size for the participants. In a paired‐samples t‐test to assess the difference between the importance and performance of nursing managers' awareness of Magnet certification, as a result of a calculating power (1−*β*) = 0.80, significance level *α* = .05 and effect size (medium) *d* = 0.5, we calculated the minimum sample number as 128 people.

We selected 10 hospitals from higher general hospitals and general hospitals with more than 500 beds. Currently, Korea does not have Magnet hospitals (ANCC, n.d.‐c) and no hospitals are preparing for Magnet certification. However, hospitals with nurse working environments that are relatively close to Magnet certification standards were targeted.

To ensure regional representation, 150 nurse managers working at 10 tertiary and general hospitals in more than four regions of Korea participated in the survey. After 10 copies with incomplete responses were excluded, 140 questionnaires remained and were used in the final analysis.

### 2.3 Instrumentation

The criteria for preparing for the Magnet Recognition Program items were based on the ANCC guidebook (American Nurses Credentialing Center, 2019). We measured content validity using a content validity index (CVI). A group of experts scored the items based on their clarity and comprehensiveness, using a rating scale ranging from 1 (none) to 4 (high level of relevance, clarity and comprehensiveness). The CVI was calculated by considering the ratio of scores of three or four for all items. If the ratio was >0.8, the item was deemed to have high content validity (Hair et al., 2009).

To confirm the perceptions of Korean nurse managers regarding Magnet designation criteria, a preliminary question was prepared by referring to the guide presented by the Magnet Recognition Program. In total, 50 preliminary items were selected and configured to simultaneously measure the degree of perception of the importance and performance of the Magnet recognition criteria. The mean CVI was 0.93. Following discussions with the expert group, the tool for Magnet certification criteria comprised 46 questions in four areas. Four items had a low CVI as they did not fit the context of Korean hospitals, and nurse managers were not able to answer them; these items were deleted. The final questionnaire comprised 46 items, including 9 items for transformational leadership (TL); 9 items for structural empowerment (SE), 21 items for exemplary professional practice (EP) and 7 items for new knowledge, innovations and improvements (NK). We conducted a reliability analysis of the final tool, and Cronbach's alpha for this study was .964 (Table 1). Additionally, to confirm the validity of the scale verified in this study, we performed exploratory factor analysis (EFA) using performance response values. The factor loading values for each classification met the 0.4 standard, thus confirming the validity of the scale without removing any items.

**TABLE 1:** Reliability analysis of the Magnet Recognition Program Questionnaire (*N* = 140).

Areas (No. of items)	Cronbach's *α*
Total (46)	.964
Transformational Leadership (9)	.863
Structural Empowerment (9)	.883
Exemplary Professional Practice (21)	.930
New Knowledge, Innovation and Improvements (7)	.909

### 2.4 Data collection

We conducted an online survey from January 21 to February 11, 2022, to collect data on nurse managers' perceptions of the importance and performance level of Magnet designation items. We first obtained approval from the Institutional Review Board (IRB No. CHAMC 2021‐12‐023‐003) and then from the nursing departments of the relevant hospitals. The nursing department of the hospital that approved the study provided a recruitment notice with a URL link to the online survey at the bottom of the email explanation. Nurse managers participated in the survey, and participants were allowed to respond only after confirming their agreement to participate. During the consent process, all study participants were informed of the purpose of the research, participation period, procedures and methods, number of research participants, expected risks and benefits to research participants, compensation for losses resulting from participation in the study, matters regarding withdrawal of consent and provision of personal information, and the researchers' contact information.

### 2.5 Data analysis

We analysed the data using SPSS WIN 25.0 (SPSS Inc., Somers, NY, USA). We evaluated the content validity of the Magnet designation criteria using the item‐content validity index (I‐CVI) and verified the reliability of the Magnet designation items using Cronbach's alpha coefficients. Additionally, we conducted an EFA using the performance results to confirm the validity of the scale used in this study.

We calculated the importance and performance of the participants' Magnet designation criteria as mean and standard deviation. Further, we analysed the difference in perception between importance and performance using a *t*‐test and an IPA matrix that included the original partitions of Martilla and James (1977) as well as one that used the transformed analytical framework by Abalo et al. (2007).

We used IPA for management and marketing strategies and to simultaneously analyse how ‘importance’ and ‘performance’ are perceived as key factors (Martilla & James, 1977, Figure 1). According to Zarei et al. (2020), to perform IPA, the factors being evaluated are first assessed based on their importance and performance ratings and then partitioned into four quadrants. To achieve this, we used a two‐dimensional grid, with the horizontal and vertical axes representing importance and performance, respectively. We plotted the factors on a grid according to their respective importance and performance ratings and identified four quadrants based on these ratings, defined as follows:

- Maintaining good work (Quadrant I): This quadrant includes factors that are highly important and have a high level of performance. These factors have already performed well and should be maintained at their current levels.
- Possible overkill (Quadrant II): This quadrant includes factors that have low importance but a high level of performance. These factors may consume resources that are better allocated to other areas.
- Low priority (Quadrant III): This quadrant includes factors of low importance and low performance. These factors may not require further improvement.
- High priority (Quadrant IV): This quadrant includes factors that are highly important and have low performance. These factors require immediate attention and should be prioritized for improvement.

**FIGURE 1:**
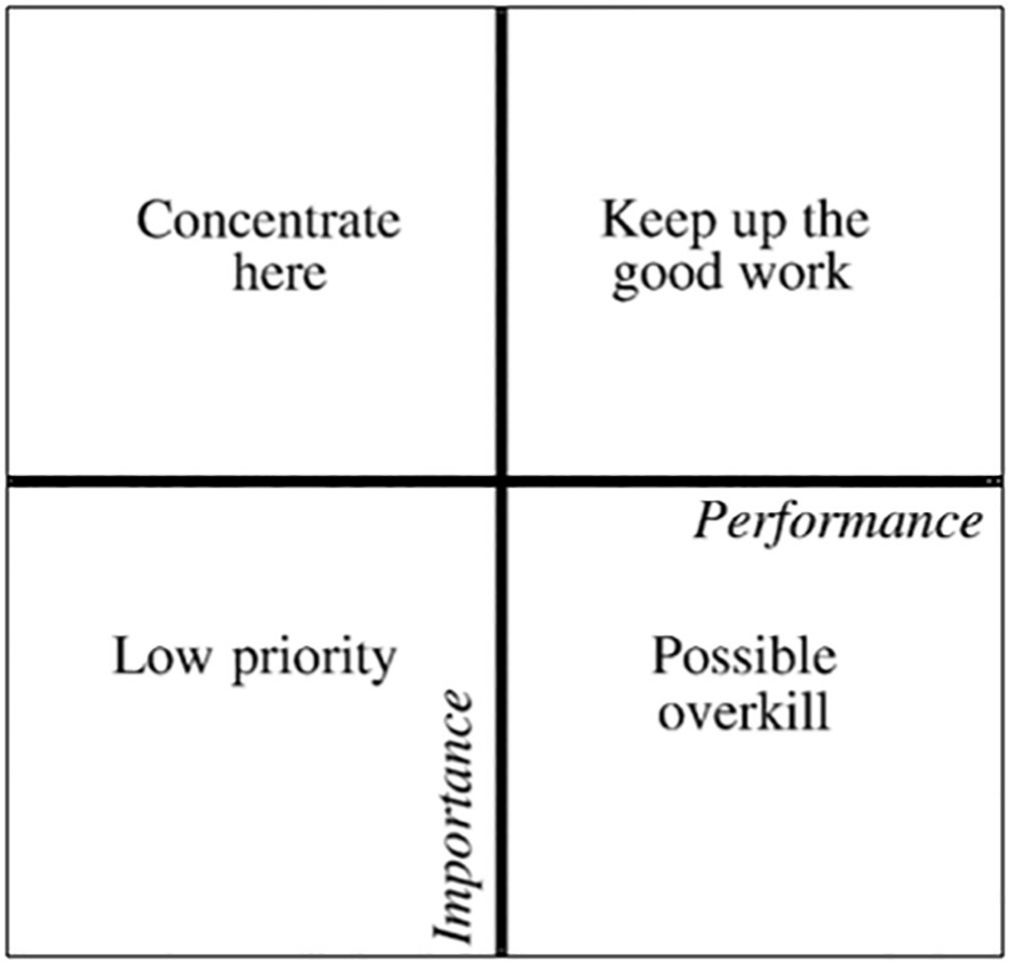
The original partitions of the IPA grid (as in Martilla JA andd James JC).

**FIGURE 2:**
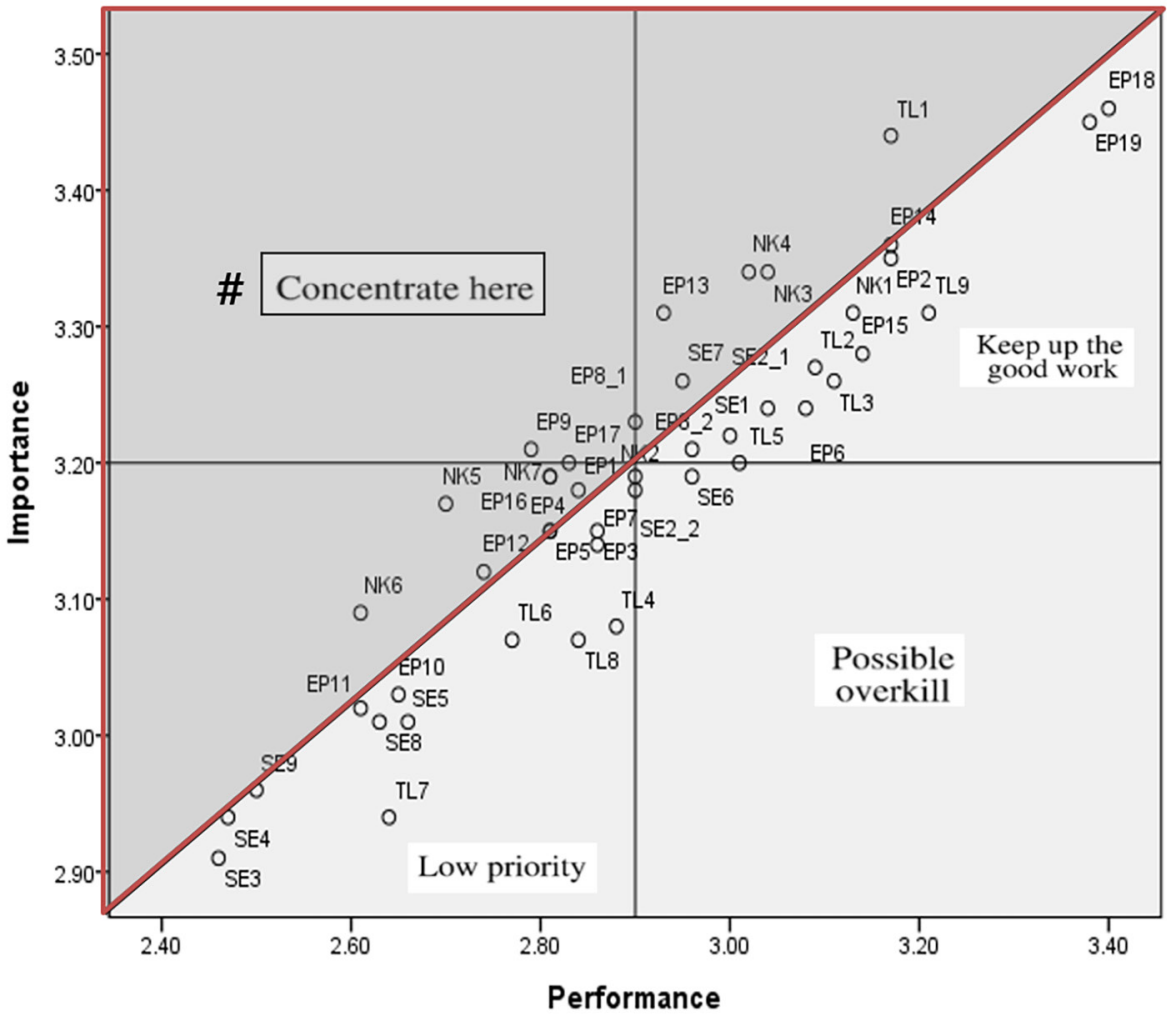
The partitions of the IPA grid (as in Abalo et al.) and IPA analysis results (Refer to Table 2 for the legend).

In this study, we used an IPA partition modified by Abalo et al. (2007). This method not only identifies item‐specific importance and performance values, but it also identifies attributes as targets for improvement measures to further emphasize the need to concentrate efforts on a particular area (Figure 2).

## 3 RESULTS

### 3.1 General characteristics of participants

Of the 150 nurse managers in the 10 hospitals, 10 provided responses that were incomplete; 140 were therefore included in the final analysis. The respondents' demographic characteristics included age, gender, education level and type of medical institution. Women accounted for 98.6% (*n* = 138); 95% (*n* = 133) were in their 40s or older and 82.1% (*n* = 115) were married. Regarding educational level, 79.3% had master's degrees (*n* = 111), 99.3% (139) were from general hospitals or higher and 87.1% (*n* = 122) were staff nurses. Regarding total clinical experience, 35% (*n* = 49) had more than 30 years, 64.3% (*n* = 90) had more than 1 year and less than 5 years of clinical experience in their current department and 45% (*n* = 63) worked in general wards in their current department.

### 3.2 Importance and performance analysis of the magnet recognition program items

The questionnaire results showed that the average importance and performance for the developed Magnet recognition criteria were 3.19 ± 135 and 2.90 ± 222, respectively. In the area of ‘transformational leadership’, the importance score was 3.18 ± 0.155 and the performance score was 2.97 ± 0.196; in ‘structural empowerment’, the importance score was 3.09 ± 0.138 and the performance score 2.76 ± 0.236; in ‘exemplary professional practice’, the importance score was 3.22 ± 0.119 and the performance score was 2.94 ± 0.219. Last, in ‘new knowledge, innovation and improvements’, the importance score was 3.23 ± 0.097 and the performance score 2.89 ± 0.191. Thus, the importance scores were higher than the performance scores in all areas (Table 2).

**TABLE 2:** Awareness of the importance and performance of the Magnet Recognition Program (*N* = 140).

Areas	Items	Importance	Performance	*t*	*ρ*
Total		3.19 ± 0.135	2.90 ± 0.222	18.023	<0.001
Transformational leadership (TL 1–9)	Total by area	3.18 ± 0.155	2.97 ± 0.196	9.385	<0.001
	Provide one example, with supporting evidence				
	1. Of an initiative in nursing practice that is consistent with the organization's mission statement^a^	3.44 ± 0.498	3.16 ± 0.530	6.872	<0.001
	2. Of an improved patient outcome associated with a goal in the nursing strategic plan	3.28 ± 0.577	3.09 ± 0.603	5.035	<0.001
	3. Of an assistant vice president's (AVP's)/a nurse director's/a nurse manager's advocacy for resources to support an organizational/unit goal	3.26 ± 0.571	3.10 ± 0.631	4.107	<0.001
	4. Of the CNO's leadership that led to a strategic organizational change	3.08 ± 0.612	2.88 ± 0.640	4.624	<0.001
	5. Of an improved patient outcome associated with an AVP's, nurse director's or nurse manager's membership in an organization‐level, decision‐making group	3.20 ± 0.527	3.01 ± 0.551	4.973	<0.001
	6. Of a mentoring plan or program for clinical nurse(s)/nurse managers/AVPs/nurse directors (exclusive of nurse managers)/advanced practice registered nurses (APRNs)/the CNO	3.07 ± 0.583	2.77 ± 0.661	6.353	<0.001
	7. Of succession‐planning activities for the nurse manager/the APRN/the AVP/nurse director/the CNO role	2.94 ± 0.628	2.64 ± 0.626	5.842	<0.001
	8. Where a clinical nurse(s) utilized data to advocate for the acquisition of a resource, in support of the care delivery system(s)	3.07 ± 0.610	2.84 ± 0.694	4.669	<0.001
	9. Of an improvement in patient care or the nursing practice environment associated with communication between the clinical nurse(s) and the CNO/the AVP/nurse director/a nurse manager	3.31 ± 0.564	3.21 ± 0.606	2.856	0.005
Structural empowerment (SE 1 ~ 9)	Total by area	3.09 ± 0.138	2.76 ± 0.236	10.287	<0.001
	Provide				
	1. Two examples, with supporting evidence, of an improved patient outcome associated with the participation of clinical nurse(s) serving as member(s) of an organization‐level interprofessional decision‐making group	3.22 ± 0.590	3.00 ± 0.648	5.142	<0.001
	2–1. One example, with supporting evidence, of an improved patient outcome associated with an evidence‐based change in nursing practice that occurred due to a clinical nurse's or clinical nurses' affiliation with a professional organization	3.24 ± 0.585	3.04 ± 0.664	4.627	<0.001
	2–2. One example, with supporting evidence, of an improved patient outcome associated with the application of nursing standards of practice implemented due to a clinical nurse's or clinical nurses' participation in a nursing professional organization	3.18 ± 0.617	2.89 ± 0.678	6.114	<0.001
	3. A description and supporting evidence of the organization's action plan for registered nurses' progress toward obtaining professional certification	2.91 ± 0.751	2.46 ± 0.783	6.727	<0.001
	4. One example, with supporting evidence, demonstrating nursing has met a targeted goal at the organizational level for improvement in professional nursing certification (e.g., percentage of nurses certified)	2.95 ± 0.777	2.46 ± 0.847	7.634	<0.001
	5. One example, with supporting evidence, of an improved patient outcome associated with knowledge gained from a nurse's or nurses' participation in a professional development activity	3.01 ± 0.673	2.66 ± 0.717	6.653	<0.001
	6. Two examples, with supporting evidence of an improved patient outcome associated with a nursing continuing education assessment and a related implementation plan	3.19 ± 0.563	2.96 ± 0.685	4.773	<0.001
	7. Evidence of a nationally accredited transition to practice program and provide one example, with supporting evidence that demonstrates the effectiveness of the transition to a practice program for new graduate nurse(s) of a newly hired experienced nurse into the nursing practice environment/a nurse transferring within the organization to a new nurse practice environment/an APRN into the practice environment/nurse managers into the new role^a^	3.26 ± 0.664	2.95 ± 0.713	6.070	<0.001
	8. One example, with supporting evidence, of the organization's recognition of a clinical nurse/a group of nurses for their contribution(s) in addressing the strategic priorities of the organization (e.g., compensation payment)	3.01 ± 0.764	2.63 ± 0.765	6.781	<0.001
	9. One example, with supporting evidence, of the organization's recognition of an interprofessional group (inclusive of nursing) for their contribution(s) in influencing the clinical care of patients (e.g., compensation payment)	2.96 ± 0.753	2.50 ± 0.785	7.832	<0.001
Exemplary professional practice (EP 1 ~ 19)	Total by area	3.22 ± 0.119	2.94 ± 0.219	11.248	<0.001
	1. Provide two examples, with supporting evidence, of an improved outcome associated with an evidenced‐based change made by clinical nurses in alignment with the organization's professional practice model (PPM)^a^	3.18 ± 0.638	2.84 ± 0.630	7.421	<0.001
	2. Present all eligible RN satisfaction data (inpatient care, ambulatory care and administrative settings)	3.36 ± 0.626	3.17 ± 0.791	3.375	0.001
	Provide one example, with supporting evidence, (No. 3–15)				
	3. Of an improvement in a patient outcome associated with one (internal or external) expert or multiple (internal or external) experts' recommended change in nursing practice	3.16 ± 0.599	2.85 ± 0.715	5.858	<0.001
	4. Of nurses' participation in interprofessional collaborative practice to ensure coordination of care across the spectrum of healthcare services	3.14 ± 0.634	2.81 ± 0.668	6.316	<0.001
	5. Of an improvement in a defined patient population outcome associated with nurse participation in an interprofessional collaborative plan of care	3.15 ± 0.613	2.81 ± 0.658	6.803	<0.001
	6. Of an improved outcome associated with an interprofessional quality improvement (QI) activity, led or co‐led by a nurse (exclusive of the CNO)/a clinical nurse	3.24 ± 0.559	3.09 ± 0.608	4.287	<0.001
	7. Of an improved patient outcome associated with an interprofessional education activity led or co‐led by a nurse (exclusive of the CNO)	3.14 ± 0.597	2.86 ± 0.683	6.140	<0.001
	8–1. Of a time when clinical nurses collaborated with an assistant vice president (AVP)/nurse director to evaluate data in order to address an identified unit‐level staffing need^a^	3.23 ± 0.650	2.90 ± 0.742	6.187	<0.001
	8–2. When nurses collaborated with an AVP/nurse director to evaluate data, in order to meet an operational need (not workforce related)	3.21 ± 0.621	2.96 ± 0.719	5.251	<0.001
	9. Of an improvement in the organization's/ clinical unit's nurse turnover rate associated with clinical nurses' participation in nursing retention activities^a^	3.21 ± 0.629	2.79 ± 0.766	6.828	<0.001
	10. Of the use of periodic formal performance review for the CNO/an AVP/nurse director/a nurse manager/an advanced practice registered nurse (APRN)/a clinical nurse that includes a self‐appraisal and peer feedback process, demonstrating a plan for professional development	3.03 ± 0.729	2.65 ± 0.822	6.184	<0.001
	11. Of clinical nurses having the authority and freedom to make nursing care decisions within the full scope of their nursing practice	3.02 ± 0.704	2.61 ± 0.726	7.345	<0.001
	12. Of nurses applying available resources to address ethical issues related to clinical practice^a^	3.12 ± 0.669	2.74 ± 0.697	6.907	<0.001
	13. Of an improved workplace safety outcome for nurses, specific to violence (e.g., physical or psychological violence, threats of incivility) toward nurses in the workplace^a^	3.31 ± 0.660	2.93 ± 0.717	6.779	<0.001
	14. of an improved patient safety outcome associated with clinical nurse involvement in the evaluation of patient safety data at the unit level	3.36 ± 0.538	3.17 ± 0.578	5.035	<0.001
	15. of a nurse‐driven initiative based on patient feedback that was received as a result of a service recovery effort	3.28 ± 0.616	3.14 ± 0.696	3.573	<0.001
	16. Provide eight of the most recent consecutive quarters of unit‐ or clinic‐level nurse‐sensitive, clinical indicator data^a^	3.19 ± 0.748	2.81 ± 0.902	6.554	<0.001
	17. Provide two nurse‐sensitive clinical indicators from the most recent eight consecutive quarters of unit‐ or clinic‐level nurse‐sensitive, clinical indicator data from the ambulatory setting (e.g., emergency department(s), ambulatory surgery center(s) and nurse‐run clinic(s))^a^	3.19 ± 0.746	2.84 ± 0.888	6.022	<0.001
	18. Provide the most recent eight consecutive quarters of inpatient satisfaction data at the unit level	3.46 ± 0.568	3.40 ± 0.666	1.745	0.083
	19. Provide the most recent eight consecutive quarters of ambulatory care setting patient satisfaction data at the unit level	3.45 ± 0.593	3.38 ± 0.676	1.745	0.083
New knowledge, innovation and improvements (NK 1 ~ 7)	Total by area	3.23 ± 0.097	2.89 ± 0.191	8.594	<0.001
	1. Provide a synopsis of one completed institutional review board‐approved (IRB‐approved) nursing research study	3.31 ± 0.723	3.13 ± 0.827	4.125	<0.001
	Provide one example, with supporting evidence, of (No. 2–7, only No. 6, two examples)				
	2. how clinical nurses disseminated the organization's completed nursing research study to internal/external audiences	3.18 ± 0.727	2.90 ± 0.848	5.289	<0.001
	3. clinical nurses' implementation of an evidence‐based practice that is new to the organization and of clinical nurses' use of an evidence‐based practice to revise an existing practice within the organization^a^	3.34 ± 0.621	3.04 ± 0.688	6.079	<0.001
	4. How clinical nurses incorporate professional specialty standards or guidelines to implement a practice new to the organization^a^	3.34 ± 0.621	3.02 ± 0.704	6.573	<0.001
	5. An innovation within the organization involving nursing^a^	3.17 ± 0.670	2.70 ± 0.739	8.054	<0.001
	6. An improved outcome in a care setting associated with a clinical nurse(s) involvement in the adoption of technology^a^	3.09 ± 0.702	2.61 ± 0.779	7.844	<0.001
	7. An improved outcome associated with nurse involvement with the design or redesign of work environment and clinical nurse involvement with the design or redesign of work flow in an ambulatory setting^a^	3.20 ± 0.628	2.81 ± 0.721	7.511	<0.001

^a^ Corresponds to the ‘Concentrate here’ area of Figure 2.

We displayed the coordinate points of the 46 items on a plane based on the x‐ and y‐axes and used the mean values of all items and performance as a split line to represent a four‐equivalent grid plot (Figure 1). The partition, which combines quadrant and diagonal‐based methods, is divided into an enlarged left quadrant (concentrated here) and diagonally underlying areas (‘Keep up the good work’, ‘Possible overkill’ and ‘Low priority’) to analyse the items located in those areas (Abalo et al., 2007). As shown in Figure 2, the items corresponding to ‘Concentrate here’ were as follows: TL 1; SE 7; EP 1, 8–1, 9, 12, 13, 16, 17 and NK 3, 4, 5, 6, 7. The items corresponding to ‘Keep up the good work’ were as follows: TL 2, 3, 9, SE 1, 2–1; EP 2, 6, 8–2, 14, 15, 18, 19 and NK 1. The one item corresponding to ‘Possible overkill’ was SE 6. Last, the items corresponding to ‘Low priority’ were as follows: TL 4, 6, 7, 8; SE 3, 4, 5, 8, 9 and EP 3, 5, 7, 10, 11. The items located on the boundary between the two areas of the partition were TL 5, SE 2–2, EP 4 and NK 2.

## 4 DISCUSSION

This study is the first step in preparing for Magnet hospital designation and is the first empirical study in Korea to investigate the importance and performance of Magnet recognition criteria according to nurse managers. Similar to many other countries, South Korea struggles to recruit and retain professional nursing personnel (Jeong et al., 2011; Kang, 2012). Introducing a Magnet‐designation hospital routine should be actively considered as it may reduce high nurse turnover (Jung & Jeong, 2018; Kim & Park, 2020; Lee & Park, 2021). To date, IPA studies on the Magnet designation criteria have been few, which makes it difficult to directly compare these results with existing studies. Nevertheless, we discuss our results.

An analysis of the differences in the importance and performance levels of the Magnet recognition criteria according to nurse managers showed that the total importance score was 3.19, which was higher than the performance score of 2.90. Additionally, the importance scores were higher than the performance scores for all 46 items; this indicates that, despite nurse managers perceiving the Magnet recognition criteria as important, their performance in the actual hospital environment was low. This result is consistent with previous studies reporting that nurse managers' management capabilities have higher importance than their performance and that a gap exists between importance and performance (Kang & Kim, 2017; Kim & Kim, 2016). Therefore, although nurse managers have a high awareness of the importance of management competencies, they cannot properly perform these competencies in actual management situations. Thus, our results highlight that to effectively prepare for Magnet hospital recognition; nurses must have working environments that can improve the performance of Magnet preparation programs.

As a result of using Abalo et al. (2007) IPA according to partition classification, the EP and NK items were the most common items in the ‘Concentrate here’ area. Items were designated in this area in case of an example/case for each of the following: nursing educational program effects, evidence‐based nursing practice, professional nursing practice models, nurses' participation in improving the turnover rate and cases of innovation in nursing. Urden et al. (2021) confirmed the perspective on Magnet redesignation through qualitative research on chief nursing officers, specifically emphasizing listening to nurses' voices, driving practice changes and monitoring continuous environmental changes. Increasing nurses' participation in hospital management (ANCC, n.d.‐b), which is also included in the Magnet hospital designation criteria, may increase their awareness of the nursing work environment and enhance their organizational commitment. In other words, it is important to create a work environment in which nurses can participate in important nursing‐related decisions. However, this is seldom the case in Korean hospitals (Lee & Park, 2021).

In total, we also identified 13 items in the ‘Keep up the good work’ area, where professionalism can be confirmed by Korean hospital nursing departments with prolonged efforts such as improved patient outcomes, participation in professional nursing organizations, and improvement of patient safety and services. General hospitals in Korea regularly undergo evaluations for medical institution certification (Kim & Kim, 2019), and preparations are usually made for matters related to patient outcomes and safety. This fact is linked to the content of SE 6 for the ‘Possible Overkill’ item. Finally, we identified 14 items in the ‘low‐priority area’, including institutional support for professional qualifications, proportion of nurses with specified certificates, cases of expertise recognition and authority and freedom to make decisions on nursing work. Although institutional support to ensure nursing professionalism is essential, nurse managers in this study viewed it as having low importance and performance. They did not recognize institutionalizing support, authority and autonomy in decision‐making to improve nurses' professionalism as high‐priority items. Further research is required to determine whether this is because the related system is well established or whether the awareness of necessity is low.

According to the IPA initial analysis framework (Martilla & James, 1977, Figure 1), ‘Concentrate here’ included only EP 9: ‘Provide one example, with supporting evidence, of an improvement in the organization's/of a clinical unit's nurse turnover rate associated with clinical nurses' participation in nursing retention activities’, which is similar to previous studies viewed from a hospital aspect (e.g., a system reform or work environment improvement) rather than nurses' participation in improving nurse turnover (Kwon & Kim, 2019). ‘Possible overkill’ included SE 6: ‘Provide two examples, with supporting evidence, of an improved patient outcome associated with a nursing continuing education assessment and a related implementation plan’. In accordance with Article 20, Paragraph 2 of the Enforcement Rules of the Medical Act, Korean nurses must be provided with continuous education, such as more than 8 h of remuneration education per year (Ministry of Health and Welfare, Korea, 2021). The questionnaire has been viewed from the perspective of completing continuous education; therefore, further research is required.

In Korea, the turnover rate of nurses remains high, and determining whether nurses are recognized for their expertise and have a working environment conducive to caring for patients is difficult (Jung & Jeong, 2018; Kim & Park, 2020; Lee & Park, 2021). To retain professional nursing personnel, their work environment must be improved. Accordingly, the Ministry of Health and Welfare announced the ‘Measures to Improve the Working Environment and Treatment of Nurses’ (Ministry of Health and Welfare, Korea, 2018). As the Magnet Recognition Program in this context focuses on nursing, it will positively impact the improvement of nurses' working environments. Therefore, hospitals should consider preparing for Magnet hospital designations rather than merely introducing them into articles. During this process, how nurses' working environments in hospitals have improved and what remains to be improved should be discussed.

### 4.1 Study strengths and limitations

This study has several limitations. First, the measurement tool used for evaluating the Magnet Recognition Program criteria was adapted for this study. Although it demonstrated good reliability and validity in this context, it may not fully capture all dimensions of the original constructs as intended by the developers. Therefore, with regard to the generalizability of the measurement tool, the findings should be interpreted with caution. Second, the cross‐sectional design of this study limits our ability to draw causal inferences from the data. The relationships observed between variables reflect associations at a single point in time, and we cannot determine the directionality or causality of these relationships. Longitudinal studies are needed to better understand the causal pathways involved. Third, we collected the data using self‐reported questionnaires, which may have introduced social desirability and recall biases. Participants may have responded in a manner that they perceived to be more socially acceptable or may have had difficulty accurately recalling past experiences. These biases may have affected the accuracy of the reported perceptions and evaluations. Despite these limitations, this study provides valuable insights into the perceptions of nursing managers regarding the Magnet Recognition Program criteria in Korean hospitals, and it lays the groundwork for future research in this area.

### 4.2 Implications for nursing practice

This study of Korean hospital nurse managers' perceptions of the Magnet Recognition Program provides practical insights for hospital administrators. In the Korean nurses' working environments identified in this study, most conditions for recognition had been established, and only a few items needed improvement. This study proposes that Korean hospitals introduce the Magnet Recognition Program and provide a theoretical basis for preparing for Magnet designation by determining nurse managers' perspectives on Magnet recognition criteria and practical difficulties before implementing the first Magnet Recognition Program in Korea.

Based on this study's results, we suggest the following. First, hospitals in Korea should actively explore the possibility of pursuing Magnet designation. This includes creating an environment consistent with Magnet principles, sharing decision‐making and highlighting professional development. Second, hospitals must provide improved educational programs, promote EBP through nursing education programs, and encourage good professional practice as these measures can improve skills and boost nurses' overall job satisfaction. Third, it is necessary to recognize and support nursing expertise, encourage participation in patient safety programs, and implement efforts, such as system improvements. Acknowledging these efforts could improve nurses' motivation and job satisfaction. Finally, we evaluated the effectiveness of systems that support existing professional qualifications and decision‐making rights. If necessary, measures should be implemented to strengthen these aspects and contribute to the full support of nurses in their roles.

## 5 CONCLUSION

We conducted this study to identify hospital nurse managers' perspectives on the Magnet Recognition Program using importance–performance analysis and provide basic data for introducing Magnet hospitals in Korea. Applying the Magnet Recognition Program in Korean hospitals will have a positive effect on the quality of nursing services, such as increasing job satisfaction for nurses and creating a better work environment. Our findings provide a foundation for strategic interventions to improve nurses' working environment in Korea. Focusing on the specific areas identified by this study will allow for maintaining a sustainable level of nursing in Korean hospitals.

## AUTHOR CONTRIBUTIONS

ER, SHJ, NYS and SY made substantial contributions to conception and design, or acquisition of data, or analysis and interpretation of data. ER and SY involved in drafting the manuscript or revising it critically for important intellectual content. ER, SHJ, NYS and SY give final approval of the version to be published. ER, SHJ, NYS and SY agreed to be accountable for all aspects of the work in ensuring that questions related to the accuracy or integrity of any part of the work are appropriately investigated and resolved.

## FUNDING INFORMATION

This work was supported by the Basic Science Research Program through the National Research Foundation of Korea (NRF), funded by the Ministry of Education (No. NRF‐2021R1F1A1062414).

## CONFLICT OF INTEREST STATEMENT

No conflict of interest has been declared by the author.

## ETHICS STATEMENT

This study has been approved (Bundang CHA Hospital IRB No. CHAMC 2021‐12‐023‐003), strictly adhering to ethical principles and protecting the privacy of respondents. Prior to participating in the study, informed consent has been obtained from each participant. Our study was based on the Helsinki Declaration.

## Data Availability

The data supporting the findings of this study are available from the corresponding author upon request.
